# Apoptosis of lens epithelial cells induced by high concentration of glucose is associated with a decrease in caveolin-1 levels

**Published:** 2009-09-30

**Authors:** Zhiyong Zhang, Ke Yao, Chongfei Jin

**Affiliations:** Eye Center, Affiliated Second Hospital, College of Medicine, Zhejiang University, Hangzhou, China

## Abstract

**Purpose:**

Lens epithelial cell (LEC) apoptosis reduces the formation of posterior capsular opacification (PCO). The involvement of caveolin-1 in the regulation of apoptosis has been previously demonstrated in epithelial cells. In this study, we investigated the relationship between caveolin-1 and apoptosis of LECs under high glucose (HG) concentrations to explore a mechanism in the formation of PCO in diabetic patients.

**Methods:**

LECs were treated with high concentrations of glucose with or without epidermal growth factor (EGF) or simvastatin in Dulbecco’s Modified Eagle’s Medium (DMEM). Induction of apoptosis was measured by flow cytometry, and the expression of caveolin-1 was examined by immunofluorescence microscopy, quantitative real-time reverse transcription polymerase chain reaction (RT–PCR), and immunoblotting.

**Results:**

The expression of caveolin-1 was decreased, and the rate of apoptosis was increased in LECs treated with increased glucose concentration and treatment duration. When simvastatin or EGF was added to HG-treated LECs, caveolin-1 levels increased and the apoptosis rate of LECs decreased. Furthermore, colocalization of caveolin-1 and phosphatidylserine (PS) on the cell surfaces of apoptotic LECs was observed by immunofluorescence microscopy.

**Conclusions:**

We observed that in HG-treated LECs, caveolin-1 expression decreased and apoptosis increased and that simvastatin or EGF promoted the proliferation of HG-treated LECs. Although the mechanisms for the formation of PCO after cataract surgery in diabetic patients are complex, our results suggest that a high concentration of glucose is not a direct cause. The observation that simvastatin inhibited the apoptosis of HG-treated LECs in its therapeutic concentration suggests that daily dosage of 3-hydroxy-3-methylglutaryl coenzyme A (HMG-CoA) reductase inhibitor used by diabetic patients may increase PCO formation.

## Introduction

Caveolin-1, a 21–24 kDa integral membrane protein, is the major component of caveolae that are flask-shaped membrane invaginations involved in multiple cellular processes such as vesicular transport, cholesterol homeostasis, and most importantly, signal transduction [1,2]. Caveolin-1 plays a role in the organization and trafficking of caveolae [3-5] and contains a scaffolding domain that enables it to bind to several signaling proteins including epidermal growth factor (EGF) receptors, sarcoma (Src)-family kinases, protein kinases C (PKCs), endothelial nitric-oxide Synthase (eNOS), and heterotrimeric G-proteins [4]. Binding of caveolin-1 to these signal proteins inhibits their activity. However, tyrosine kinase-mediated phosphorylation of caveolin-1 abolishes this inhibition and leads to cell transformation and proliferation [6]. Caveolin-1 is present in lens epithelial cells (LECs) [7-10] and plays a pivotal role in many cellular processes such as epithelial-mesenchymal transdifferentiation (EMT) [11] and cell-to-cell communications [12].

Posterior capsular opacification (PCO) reflects the wound-healing process of LECs in the capsular bag after cataract surgery and is caused by the proliferation and EMT of LECs [13,14]. Recent data have shown that blocking the growth of LECs after cataract extraction surgery by inducing LEC apoptosis can prevent the formation of PCO [15]. Caveolin-1 has been shown to be involved in the regulation of apoptosis in macrophages [16], fibroblasts, and epithelial cells [17,18]. In the present study, we showed that high glucose (HG) concentrations in culture decreased the expression of caveolin-1 and caused LEC apoptosis while EGF, which usually increases in aqueous humor after cataract surgery, and simvastatin, an 3-hydroxy-3-methylglutaryl coenzyme A (HMG-CoA) reductase inhibitor commonly used in diabetic patients, enhanced the expression of caveolin-1 and reduced apoptosis in HG-treated LECs. The results suggest that the formation of PCO in diabetic patients after cataract surgery may not be directly caused by high concentrations of glucose but by other factors such as surgery.

## Methods

### Materials

Human lens epithelial B3 (HLE-B3) cells were obtained from ATCC (Rockville, MD). Dulbecco’s Modified Eagle’s Medium (DMEM) and fetal bovine serum (FBS) were obtained from Gibco (Grand Island, NY). Anti-caveolin-1 polyclonal antibody was purchased from BD Biosciences Transduction Laboratories (San Diego, CA). TRIZOL reagent, Vybrant^®^ apoptosis assay kit #2, and rhodamine (TRITC)-conjugated goat anti-mouse immunoglobulin G (IgG) were from Invitrogen Life Technologies (Carlsbad, CA). Anti-mouse IgG horseradish peroxidase (HRP) antibody was from Santa Cruz (Santa Cruz, CA). Polymerase chain reaction (PCR) and RNA extraction kits were from Takara Co. Ltd. (Ohtsu, Japan). EGF was from Sigma (St. Louis, MO). Simvastatin was a gift from Zhejiang Medicine Co. Ltd., Xingchang Pharmaceutical Factory (Zhejiang, China). The chemiluminescence (ECL) detection kit was from Amersham Pharmacia (Arlington Heights, IL).

### Cell culture

HLE-B3 cells were cultured in DMEM supplemented with heat-inactivated (56 °C, 0.5 h) 10% FBS at 37 °C in a humidified atmosphere with 5% CO_2_. To perform experiments, the cells (70%–80% confluent) were seeded in a 60-mm culture dish (Falcon; Becton-Dickinson, Oxnard, CA), incubated with media containing 0.1% FBS (to prevent slow apoptosis due to serum starvation) for 24 h, and then treated with freshly prepared glucose (5–25 mM), a combination of mannitol (20 mM) and glucose (5 mM; as a control to exclude the effect of osmotic pressure), simvastatin (10 nM), and EGF (0.1 ng/ml) in DMEM. At indicated time points, the cells were collected for various assays.

### Flow cytometry of apoptosis

Apoptosis was assessed by measuring membrane redistribution of phosphatidyl serine (PS) using the Vybrant^®^ apoptosis assay kit #2, which contains propidium iodide (PI) and annexin V-conjugated Alexa Fluor 488 dye [19,20]. Cells were grown on a six-well plate at 1×10^6^ cells per well and treated with glucose (5–25 mM) or mannitol (20 mM) and glucose (5 mM) in DMEM for the indicated time. To examine the effects of simvastatin or EGF on apoptosis, cells were incubated with glucose (5 or 25 mM) in DMEM containing simvastatin (10 nM) or EGF (0.1 ng/ml) for 48 h. After the drug treatment, cells were collected, washed twice with PBS, and resuspended in 500 μl binding buffer containing annexin V antibody (5 μl) and PI (5 μl of 250 μg/ml stock solution). After incubation on ice in the dark for 30 min, cells were evaluated with a fluorescence-activated cell sorting (FACS) calibur flow cytometer (Becton Dickinson, San Jose, CA). Basal apoptosis and necrosis were excluded, and the percentage of cells undergoing apoptosis was determined by three independent experiments. During the process, cell lysis rates were less than 0.5%.

### Quantitative real-time RT–PCR of caveolin-1 mRNA

Total RNA was isolated from HLE-B3 cells with TRIZOL reagent according to the manufacturer’s protocol and used as templates to generate cDNA [21,22]. The caveolin-1 (*CAV1*) mRNA levels were quantified by real-time reverse transcription polymerase chain reaction (RT–PCR). Primers were designed using the Primer Premier 5 software (Premier Biosoft International, Palo Alto, CA) [23]. The following primers were used for *CAV1* and *β-actin* amplifications: CAV1 (NM_001753) forward 5′-AAC GAT GAC GTG GTC AAG ATT G-3′ and reverse 5′-TCC AAA TGC CGT CAA AAC TGT −3′ (amplifying a 301 bp fragment) and β-actin forward 5′-CAT CAC CAT TGG CAA TGA GC-3′ and reverse 5′-TCG TCA TAC TCC TGC TTG C-3′ (amplifying a 351 bp fragment). Primers were designed to include sequences from the extremes of two exons to ensure the detection of the corresponding transcript, avoiding amplification of genomic DNA. Reactions were performed in 96-well plates with Optical-Quality eight-tube strips (Bio-Rad Laboratories, Hercules, CA). PCR was performed using SYBR Green I as the reporter dye. Increases in fluorescence were measured real time during the extension step. The cDNA was amplified under the following conditions: 95 °C for 10 min followed by 45 cycles for 15 s at 95 °C and 1 min at 59 °C. The C_t_ value of each condition was the average of triplicate runs.

### Western blotting for caveolin-1

Proteins were extracted from HLE-B3 cells as described previously [16], separated by 15% SDS–PAGE, and transferred to polyvinylidene fluoride (PVDF) membranes. After blocking with Tris-buffered saline containing Tween-20 (TBST; 20 mM Tris, 137 mM NaCl, 0.4% Tween-20, pH 7.6) and 5% non-fat dried milk, the membranes were incubated with primary antibodies in TBST containing 1% non-fat dried milk at 4 °C for 16 h. Secondary antibodies in TBST containing 1% non-fat dried milk were then added for 2 h at room temperature. Bound secondary antibodies were detected using an enhanced chemiluminescence (ECL) detection system (Amersham Pharmacia Biotech). Changes in caveolin-1 protein levels were normalized to β-actin levels in each sample.

### Immunofluorescence microscopy of caveolin-1 and phosphatidylserine

Cells were seeded on coverslips for 24 h. After being treated with or without simvastatin (10 nM) or EGF (0.1 ng/ml) in 25 mM glucose solution for 48 h, cells were fixed with paraformaldehyde for 10 min at room temperature and incubated with 0.1% Triton X-100 on ice for 5 min. To determine the colocalization of caveolin-1 and phosphatidylserine (PS) in HLE-B3 cell membrane and to analyze PS in apoptotic cells, cells treated with 25 mM glucose for 48 h were incubated with 5 μl of Alexa Fluor 488-conjugated annexin V in 100 μl of binding buffer (10 mM HEPES, 140 mM NaCl, 2.5 mM CaCl, pH 7.4) for 15 min at room temperature before being fixed with paraformaldehyde for caveolin-1 staining. To visualize caveolin-1 in cells, the coverslips were washed with PBS and sequentially incubated at room temperature in PBS containing goat serum, anti-caveolin-1 primary antibody at 1:200 dilution, and fluorescence-tagged secondary antibody for 1 h each. The cells were then washed twice with PBS, mounted on glass slides, and examined using a Leica fluorescence microscope (Heidelberg, Germany).

### Examination of caveolae by transmission electron microscopy 

HLE-B3 cells were grown on a six-well plate at 1×10^6^ cells per well and treated as described above. Cells were then fixed with glutaraldehyde, post-fixed with osmium tetroxide, stained with uranyl acetate and lead citrate, and examined using a Philips 410 transmissions electron microscope (TEM; Eindhoven, Netherlands) as described previously [24,25].

### Statistical analysis

The results are presented as mean±standard error (SE). Differences between groups were assessed by one-way analysis of variance (ANOVA) followed by post-hoc test with the S-N-K method. Calculations were performed using SPSS for Windows version 13.0 statistical package (SPSS, Chicago, IL). p values less than 0.05 were considered statistically significant.

## Results

### Effects of high glucose concentrations on apoptosis and caveolin-1 expression in HLE-B3 cells

**Figure 1:**
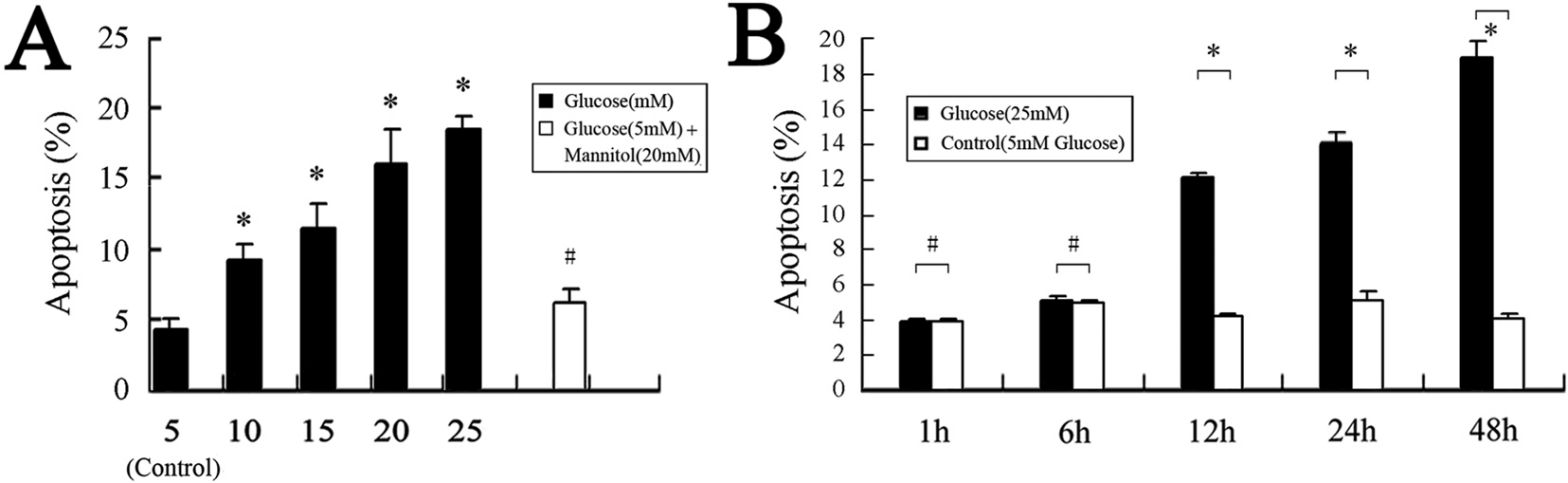
Effects of high concentrations of glucose on apoptosis of HLE-B3 cells. Cells were incubated with 5–25 mM glucose for 48 h (**A**) or with 25 mM glucose for 1–48 h (**B**). Cells treated with 5 mM glucose and 20 mM mannitol were used as an osmotic control. Apoptosis was determined by using the Vybrantan® apoptosis assay kit #2 and by flow cytometry. Experiments were performed in triplicate. The asterisk indicates p<0.05 versus control, and the hash mark denotes p>0.05 versus control

**Figure 2:**
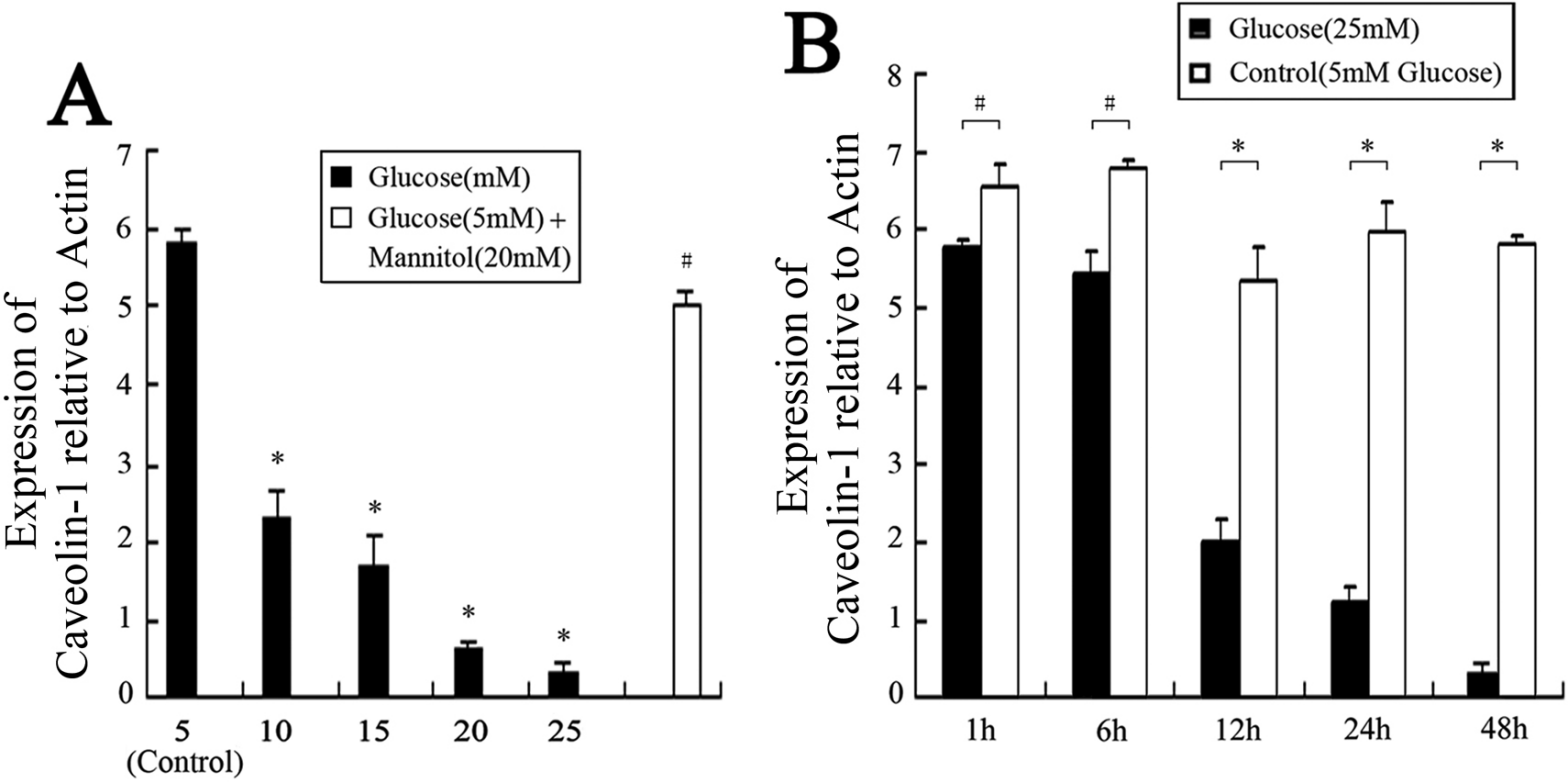
Caveolin-1 (*CAV1*) mRNA levels in HLE-B3 cells treated with high concentrations of glucose. Cells were treated with the indicated concentrations of glucose in DMEM for 48 h (**A**) or with 5 mM (control) or 25 mM glucose in DMEM for 1–48 h (**B**). *CAV1* mRNA levels were determined by real-time RT–PCR and normalized to those of β-actin. Cells treated with 5 mM glucose and 20 mM mannitol were used as an osmotic control. The experiments were performed in triplicate. **A**: The asterisk indicates p<0.01 versus control, and the hash mark denotes p>0.05 versus control. **B**: The asterisk indicates p<0.05, and the hash mark denotes p>0.05.

**Figure 3:**
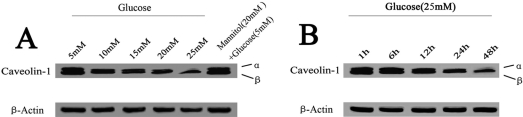
Western blot analysis of caveolin-1 in HLE-B3 cells treated with high concentrations of glucose. Cells were treated with the indicated concentrations of glucose in DMEM for 48 h (**A**) or 25 mM glucose in DMEM for 1–48 h (**B**). Cells treated with 5 mM glucose and 20 mM mannitol were used as an osmotic control. The experiments were performed in triplicate.

Based on previous reports [26,27], we cultured HLE-B3 cells with various concentrations (5–25 mM) of glucose for various lengths of time (1–48 h) to evaluate dose and time responses of cells to glucose. As described previously, 5 mM glucose represents the normal physiologic level and 25 mM the high level [26,27]. As shown in Figure 1A, 4.13%±0.74%, 9.12%±1.19%, 11.42%±1.68%, 16.00%±2.47%, and 18.53%±1.01% increases in the apoptosis rate were observed in cells treated with 5, 10, 15, 20, and 25 mM glucose for 48 h, respectively. A significant decrease in *CAV1* mRNA levels was also observed in cells treated with 5–25 mM of glucose (Figure 2A). Cells treated with 25 mM glucose had the lowest *CAV1* mRNA levels. In agreement with the RT–PCR data, decreased levels of caveolin-1 protein were detected in cells treated with 10–25 mM glucose for 48 h compared to the control (Figure 3A). After being incubated with 25 mM glucose from 1 h to 48 h, an increase in apoptosis was observed from 4.01%±0.08% at 1 h to 14.03%±0.52% at 24 h, reaching a maximum of 18.53%±1.01% at 48 h (Figure 1B). However, it was found that longer treatment time resulted in a lower level of *CAV1* expression (Figure 2B). The protein levels of caveolin-1 were also decreased in cells treated with 25 mM glucose for 12–48 h (Figure 3B). Based on these results, we selected 25 mM as the high glucose concentration and 48 h as the treatment time for subsequent studies.

Cells treated with HG (25 mM) for 48 h exhibited a significantly higher apoptosis rate and less caveolin-1 expression than those of the control (treated with 5 mM glucose). Furthermore, the addition of mannitol as an osmotic control did not influence the apoptosis rate or the expression of caveolin-1. These results indicate that apoptosis in HLE-B3 cells after HG exposure was associated with decreased expression of caveolin-1.

### Effects of HG on subcellular localization of caveolin-1 and phosphatidylserine in HLE-B3 cells

**Figure 4:**
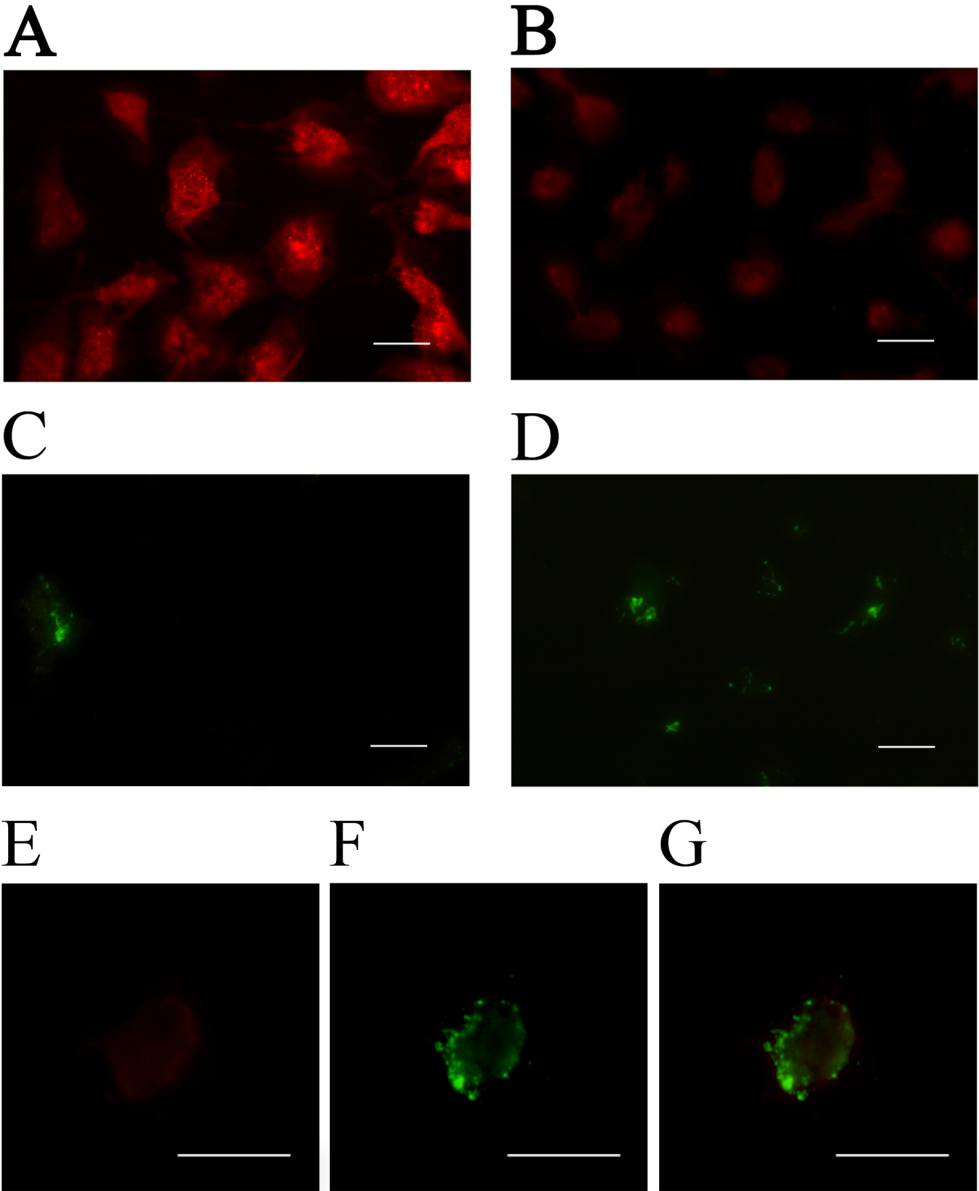
Subcellular distribution of caveolin-1 and externalization of phosphatidylserine in HLE-B3 cells treated with high concentrations of glucose. HLE-B3 cells were treated with 5 mM glucose (**A**, **C**) or 25 mM glucose (**B**, **D**, **E**, **F**) for 48 h and then reacted with anti-caveolin-1 polyclonal primary antibody (1:200; **A**, **B**, **E**) or Alexa Fluor 488-conjugated annexin V (**C**, **D**, **F**). Cells were examined with an immunofluorescent microscope. **G** is a merged image of **E** and **F**, indicating colocalization of caveolin-1 and PS in the cell membrane. The scale bars indicate 15 μm.

**Figure 5:**
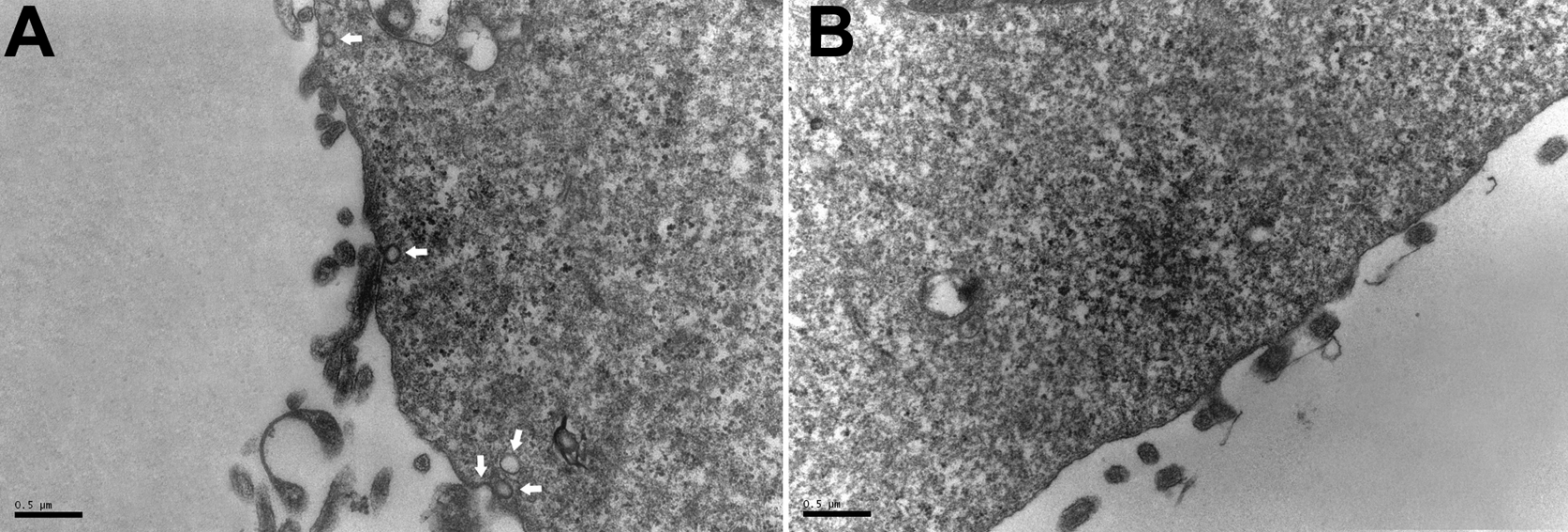
Transmission electron microscopy of caveolae in HLE-B3 cells treated with high concentrations of glucose. Cells were seeded on glass coverslips, treated with 5 mM or 25 mM glucose for 48 h, fixed with glutaraldehyde, post-fixed with osmium tetroxide, and stained with uranyl acetate and lead citrate. **A**: 5 mM glucose-treated (control) cells. Arrows point to caveolae. **B**: 25 mM glucose-treated cells.

To examine whether the change in caveolin-1 protein expression is related to HG-induced apoptosis, we immunostained the control and HG-treated cells for caveolin-1 and PS (Figure 4). Caveolin-1 fluorescence intensity was relatively high in control cells (Figure 4A) but was greatly reduced in cells treated with 25 mM glucose (Figure 4B), indicating a decrease in caveolin-1 levels in HG-treated HLE-B3 cells. A significant increase in the number of double-stained cells was also observed in HG-treated HLE-B3 cells (Figure 4C,D). As caveolin-1 and PS were both present on the cell surface, the decrease in caveolin-1 expression and the increase in PS externalization suggested a role of caveolin-1 in the apoptotic process. Therefore, we further examined whether caveolin-1 distribution in apoptotic cells resembled that of PS on the cell surface by staining HG-treated HLE-B3 cells for both caveolin-1 and PS. After being treated with 25 mM glucose for 48 h, PS was found to be translocated from the inner plasma membrane to the outer membrane and was associated with caveolin-1 (Figure 4E,F,G). Since caveolin-1 is the major component of caveolae, a decrease in caveolin-1 may reduce caveolae formation. To further characterize the changes of caveolin-1 in HG-treated HLE-B3 cells, the distribution of caveolae in cells was analyzed by transmission electron microscopy (Figure 5A,B). As expected, the number of caveolae was greatly reduced in cells treated with 25 mM glucose (Figure 5B).

### Effect of increased caveolin-1 expression on apoptosis of HG-induced HLE-B3 cells

**Figure 6:**
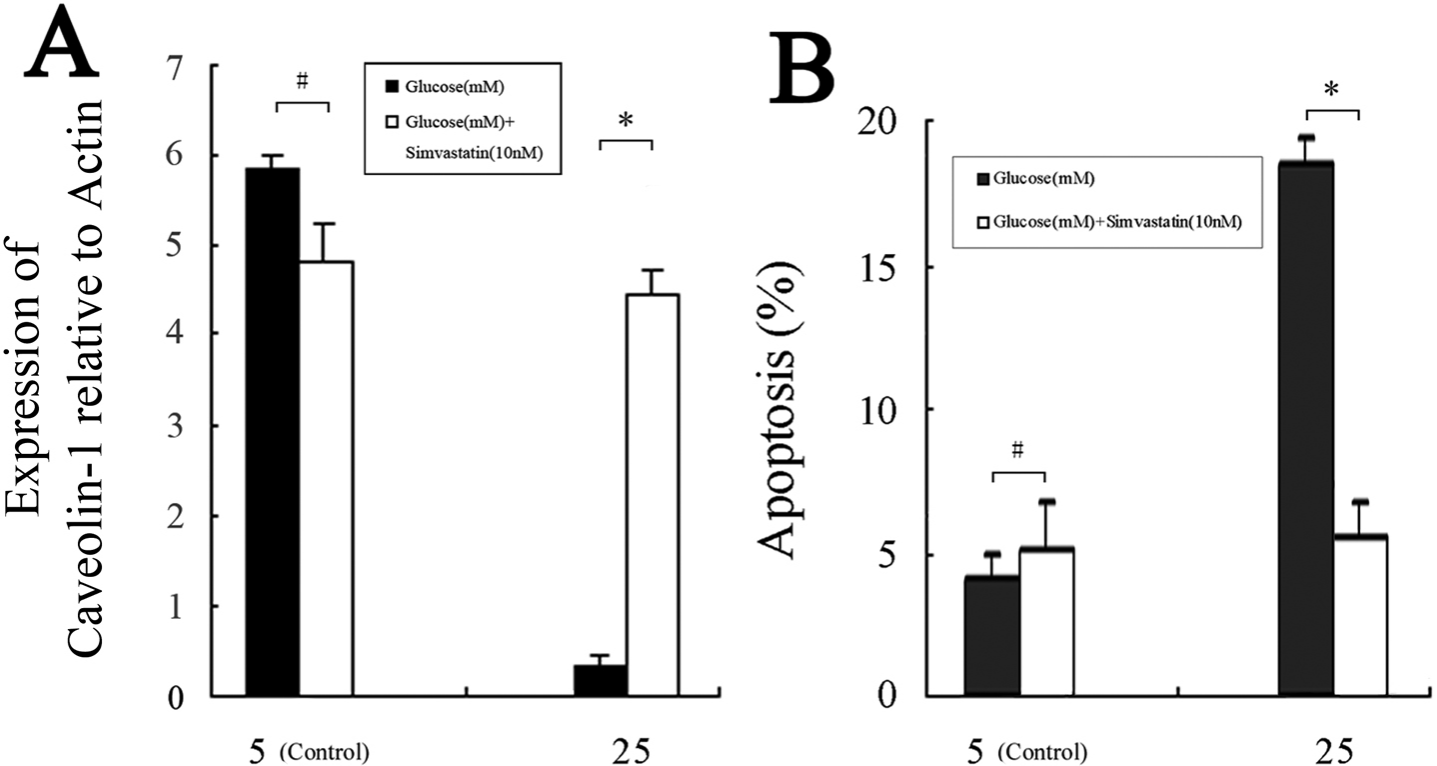
Effects of simvastatin on caveolin-1 expression and apoptosis in HLE-B3 cells treated with high concentrations of glucose. Cells were treated with simvastatin (10 nM) and glucose (5 or 25 mM) for 48 h. The ∆C_t_ values of *CAV1* mRNA levels (**A**) and the percentages of apoptotic cells (**B**) were determined by real-time RT–PCR and flow cytometry, respectively. Experiments were performed in triplicate. **A**: The asterisk indicates p<0.01, and the hash mark denotes p>0.05. **B**: The asterisk indicates p<0.05, and the hash mark denotes p>0.05.

Previous studies have shown that caveolin-1 in conjunction with HMG-CoA reductase plays an important role in cellular cholesterol homeostasis [16,28,29]. In addition, the *CAV1* mRNA levels can be regulated under conditions where the cellular cholesterol level is altered [30]. To investigate the effect of HMG-CoA reductase inhibition by simvastatin on caveolin-1 expression, HLE-B3 cells in culture media containing 5 mM or 25 mM glucose were incubated for 48 h with 10 nM of simvastatin, which represents the clinical therapeutic concentration in diabetic patients [31,32]. Results of real-time RT–PCR and flow cytometry showed no significant changes in *CAV1* mRNA levels and apoptosis in cells treated with 5 mM glucose and with or without simvastatin stress (Figure 6A,B). However, the *CAV1* mRNA levels in HLE-B3 cells treated with 25 mM glucose and 10 nM simvastin increased (Figure 6A), and the percentages of apoptotic cells decreased from 18.53%±1.01% to 5.52%±1.22% when compared to the control (Figure 6B).

**Figure 7:**
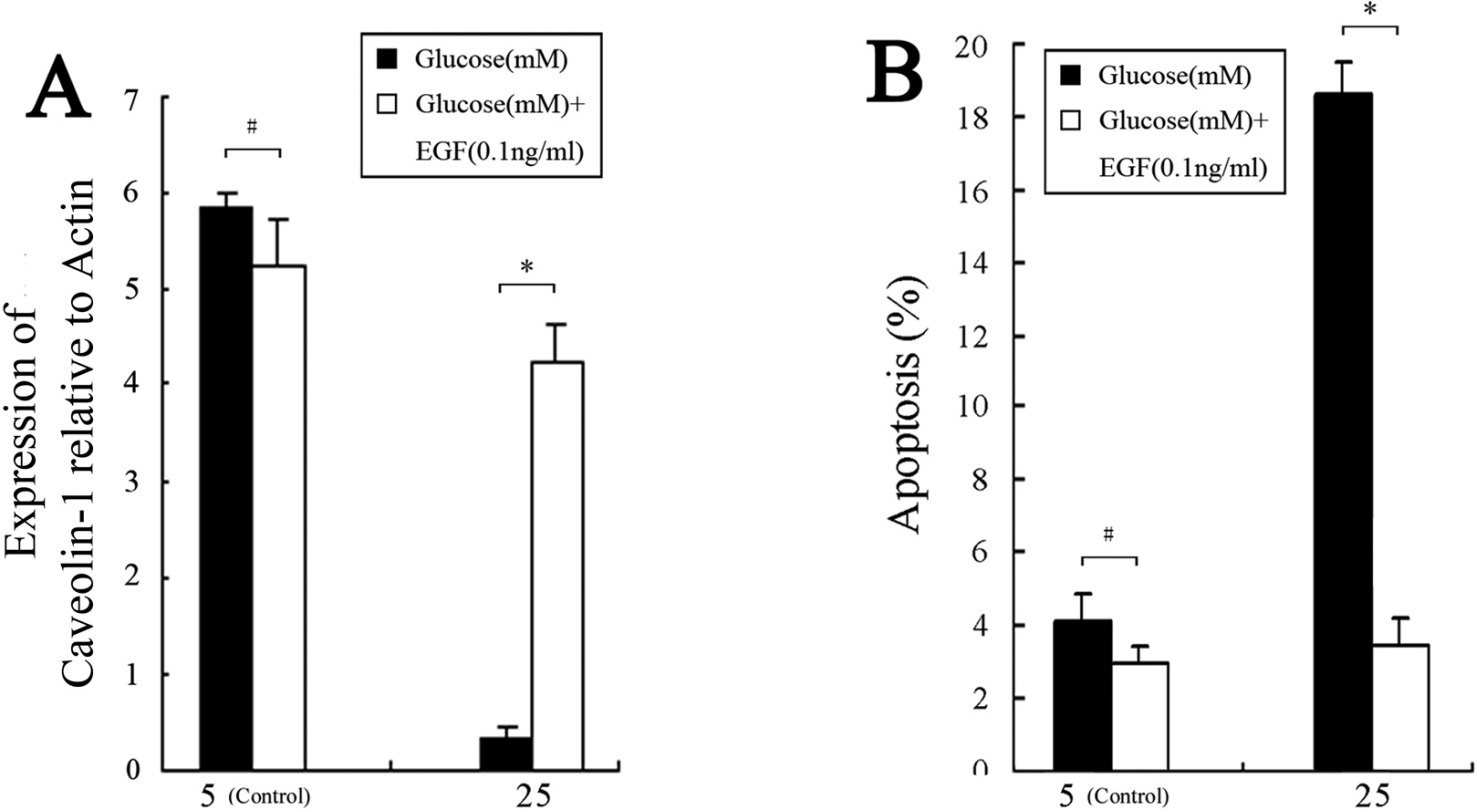
Effects of EGF on caveolin-1 expression and apoptosis in HLE-B3 cells treated with high concentrations of glucose. Cells were incubated in DMEM containing both glucose and EGF at the indicated concentrations for 48 h. The ∆C_t_ values of *CAV1* mRNA levels (**A**) and the percentages of apoptotic cells (**B**) were determined by real-time RT–PCR and flow cytometry, respectively. Experiments were performed in triplicate. **A**: The asterisk indicates p<0.01, and the hash mark denotes p>0.05. **B**: The asterisk indicates p<0.05, and the hash mark denotes p>0.05.

**Figure 8:**
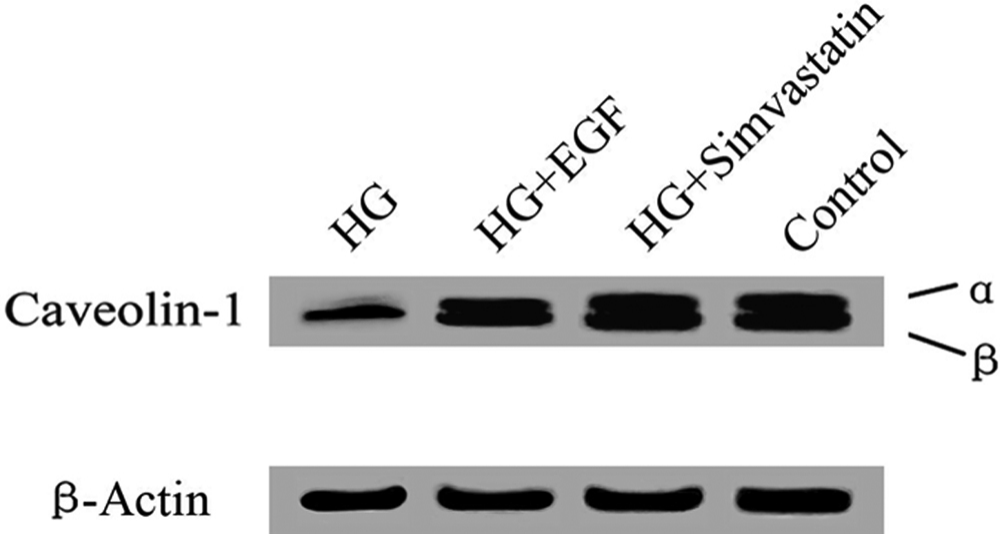
Effects of simvastatin and EGF on the levels of caveolin-1 protein in HLE-B3 cells treated with high concentrations of glucose. Cells were incubated with 5 mM or 25 mM glucose in the presence or absence of either 10 nM simvastatin or 0.1 ng/ml EGF for 48 h. The levels of caveolin-1 protein were analyzed by western blot. The experiments were performed in triplicate. HG: 25 mM glucose; Control: 5 mM glucose.

EGF has been shown to activate the phosphorylation of caveolin-1 [6] and influence caveolae assembly on the cell surface [33]. To examine the effect of EGF on caveolin-1 expression, we treated HLE-B3 cells with 5 or 25 mM glucose and 0.1 ng/ml EGF [34] for 48 h. Compared to the HG-treated control, treatment with 25 mM glucose and 0.1 ng/ml EGF significantly increased *CAV1* mRNA levels and decreased apoptosis by 3.21%±0.87% (Figure 7A,B). Similarly, western blot analysis showed that HG incubation reduced caveolin-1 protein levels (Figure 8). The addition of 0.1 ng/ml EGF or 10 nM simvastatin in 25 mM glucose notably increased caveolin-1 protein levels (Figure 8) compared to control cells that were treated only with 25 mM glucose. These results indicate that increased *CAV1* expression due to EGF or simvastatin stimulation was related to the inhibition of HLE-B3 cell apoptosis under high concentrations of glucose.

## Discussion

Caveolae, the sphingomy-cholesterol enriched microdomains, form a stable lipid matrix that act as an ordered support for receptor-mediated signaling events. It has been proposed that caveola composition changes in response to adverse extracellular conditions [35,36]. Consistent with this hypothesis, we found in the current study that HG treatment led to a decrease in the number of caveolae on the surface of LECs. We also found reduced levels of caveolin-1 mRNA and protein in HG-treated LECs, indicating that caveolin-1 expression was down-regulated at the transcription level.

Although it has been proposed that caveolin-1 functions as a message center that compartmentalizes anti- and pro-apoptotic signaling molecules on the cell surface to regulate apoptosis [17], its role in regulating apoptosis remains controversial. In the current study, we found the levels of caveolin-1 reduced and apoptosis rate increased in HG-treated LECs, indicating that caveolin-1 may inhibit apoptosis. However, overexpression of caveolin-1 in fibroblasts and epithelial cells has been shown to sensitize these cells to staurosporine-induced apoptosis, possibly via inhibition of phosphoinositide 3-kinase (PI3K) and/or activation of caspase-3 [17,18]. The hypercellularity of the lung parenchyma and the increased proliferative phenotype of mouse embryo fibroblasts from caveolin-1 knockout mice are consistent with the anti-proliferative properties of caveolin-1 [37,38]. In contrast, Timme et al. [39] showed that overexpression of caveolin-1 maintained Akt in an active state in prostate cancer cells by inhibiting protein phosphatase activity, a mechanism that may contribute to cellular resistance to c-myc-induced apoptosis. This discrepancy suggests that the involvement of caveolin-1 in the regulation of proliferation or apoptosis may not be cell-specific but rather dependent on cellular events.

In HG-treated LECs, simvastatin was found to cause an increase in the expression of caveolin-1 and inhibition of LEC apoptosis. Similarly, it has been reported that simvastatin upregulates caveolin-1 expression in human smooth muscle cells [40] and macrophages [15] and normalizes the vascular cell phenotype in severe pulmonary hypertension [41]. However, Lecian et al. [29] found that simvastatin decreases caveolin-1 expression in Madin-Darby canine kidney cells. Recent reports have demonstrated that caveolin-1 binds cholesterol, and cholesterol is thought to intercalate under the sphingolipid head groups, thus facilitating tighter packing and decreasing entropy [42]. The agents that can change cholesterol concentrations within caveolae have been shown to regulate caveolin-1 expression and alter cellular signaling [43,44]. We, therefore, speculated that the contribution of simvastatin in the regulation of caveolin-1 expression may be cell-specific and related to simvastatin effects beyond lipid lowering [45].

EGF was also found to increase caveolin-1 expression and inhibit apoptosis in HG-treated LECs. A large body of evidence has shown that caveolin-1 interacts with epidermal growth factor receptor (EGFR). Tencer et al. [46] reported that upregulation of caveolin-1 by EGF activates EGFR. In addition, overexpression of caveolin-1 in the MCF-7 breast cancer cell line has been shown to modulate EGFR activation levels and EGF-induced EGFR signaling [47]. It has also been shown that EGF regulation contributes to the stability of *CAV1* mRNA [48,49]. Since HT-29 human colon cancer cells are not sensitive to EGF stimulation due to the lack of a canonical peroxisome proliferator-activated receptor-gamma response element (PPRE) [46], we speculated that EGF may regulate caveolin-1 expression through peroxisome proliferator-activated receptor-gamma.

We also found that caveolin-1 was present on the surface of HG-treated LECs and colocalized with PS in the outer membrane of apoptotic LECs. Our results suggest that PS externalization is related to the presence of caveolin-1 in the cell membrane and the regulatory elements involved in PS externalization in apoptotic LECs under HG conditions. Previous reports indicate that store-operated Ca^2+^ entry regulates externalization of PS on the cell surface and that PS exposure is dependent in part on the extracellular signal-regulated kinase pathway associated with caveolin-1 [50]. Further investigations to corroborate the relationship between caveolin-1 and store-operated Ca^2+^ entry in HG-treated LECs are required.

Uncontrolled diabetes mellitus (DM) may lead to systemic and ocular complications including cataracts [51-54]. Although cataract surgery has been greatly improved due to the development of phacoemulsification, posterior capsular opacification remains the major complication in diabetic patients [55-57]. However, the finding that LEC apoptosis has effects on PCO in diabetic patients after cataract surgery is still controversial. Zaczek et al. [57] found less PCO occurrence in diabetic patients than in non-diabetic patients after phacoemulsification surgery and speculated that damage to LECs and the decreased proliferative activity of the damaged LECs may reduce PCO in diabetic patients under hyperglycemic conditions. However, Hayashi et al. [58] reported greater PCO occurrence in diabetic patients than in non-diabetic patients and suggested that the disruption of the blood-aqueous barrier and inflammatory reaction after cataract surgery is more severe in the eyes of diabetic patients. Since the mechanism regulating the proliferation and epithelial-mesenchymal transdifferentiation of LECs is not completely understood, the reason for the discrepant findings in PCO formation between diabetic and non-diabetic patients remains unclear.

In the current study, we observed that high concentrations of glucose inhibited LEC proliferation in culture and that simvastatin and EGF promoted LEC growth. We, therefore, speculate that high concentrations of glucose may not be a direct cause for the formation of PCO in diabetic patients. The higher incidence of PCO after cataract surgery in diabetic patients could be due to escalated chemical mediators such as interleukin-1 (IL-1), IL-6, IL-8, and EGF resulting from the breakdown of the blood-aqueous barrier after surgery. These chemical mediators may lead to an increased proliferation of LECs [59]. Our observation that simvastatin inhibited LEC apoptosis in its therapeutic concentration suggests daily dosage of HMG-CoA reductase inhibitor used by diabetic patients may increase PCO formation.

In summary, we found that high concentration of glucose decreased caveolin-1 expression and increased the apoptosis rate of LECs and that simvastatin and EGF promoted the proliferation of LECs under a high concentration of glucose. Although the mechanisms for the formation of PCO after cataract surgery in diabetic patients are complex, our results suggest that high concentration of glucose is not a direct cause.
